# Supplementary material for: Spatially structured eco-evolutionary dynamics in a host-pathogen interaction render isolated populations vulnerable to disease
Source: Nat Commun. 2022 Oct 13;13:6018. doi: 10.1038/s41467-022-33665-3 (PMC9561709; doi:10.1038/s41467-022-33665-3)
Supplement: Supplementary file 1 — Supplementary Information [file 41467_2022_33665_MOESM1_ESM.pdf]

Supplementary tables and figures for:

Spatially structured eco-evolutionary dynamics in a host-pathogen interaction render isolated populations vulnerable to disease

Layla Höckerstedt[1,2*], Elina Numminen[1*], Ben Ashby[3,4*], Mike Boots[3,5], Anna Norberg[6] and Anna-Liisa Laine[1,6]

*These authors contributed equally

[1]Organismal and Evolutionary Biology Research Program, Faculty of Biological and Environmental Sciences, 00014 University of Helsinki, Finland

[2]Current adress: Finnish Meteorological Institute, FI-00101 Helsinki, Finland

[3]Department of Integrative Biology, University of California, Berkeley, CA, 94720 USA

[4]Department of Mathematical Sciences, University of Bath, Bath, BA2 7AY, UK
[5]Biosciences, University of Exeter, Penryn, TR10 9EZ, UK

[6]Department of Evolutionary Biology and Environmental Studies, University of Zürich CH-8057 Zurich, Switzerland
Corresponding author: anna-liisa.laine@uzh.ch

Supplementary Table 1. **Spatio-temporal model of the changes in population size of *Plantago lanceolata*.** Posterior means and quantiles for the fixed effects of the model, as well as for the parameters related to the spatiotemporal random field, elaborated in equations 1-2 (M&M: *Spatio-temporal model of the changes in the population size*).

	mean	sd	0.025quant	0.5quant	0.975quant	mode	kld
Rainfall_July	-0.0103	0.0372	-0.0833	-0.0103	0.0627	-0.0103	0
Rainfall_August	0.0278	0.0461	-0.0627	0.0278	0.1183	0.0278	0
PresenceLowConnectivity	0.3455	0.2146	-0.0758	0.3455	0.7666	0.3455	0
PresenceHighConnectivity	0.6699	0.274	0.1319	0.6699	1.2074	0.6699	0
PresenceIntermediateConnectivity	0.6592	0.1389	0.3865	0.6592	0.9317	0.6592	0
AbsenceLowConnectivity	0.7763	0.0791	0.621	0.7763	0.9314	0.7763	0
AbsenceHighConnectivity	0.9816	0.0971	0.791	0.9816	1.1721	0.9816	0
AbsenceIntermediateConnectivity	0.814	0.0623	0.6918	0.814	0.9361	0.814	0
LogPlant.drynessPrev	-0.3812	0.0258	-0.4318	-0.3812	-0.3306	-0.3812	0
LogPlant.dryness	-0.0118	0.0249	-0.0607	-0.0118	0.0371	-0.0118	0
temporal autocorrelation rho:	-0.3518		-0.4376		-0.2414		
marginal variance of the spatiotemporal field:	1.41402		1.19913		1.62897		
range:	8361.564		7022.448		9958.689		

Supplementary Table 2. The data on pathogen presences and absences, after excluding the outliers. The first modelled change is change in population size from 2001 to 2002.

Year	absence (0)	presence (1)
2002	3695	125
2003	2967	141
2004	3077	30
2005	3062	71
2006	3709	107
2007	3197	207
2008	3451	203

Supplementary Table 3. Metapopulation simulation model parameters and values.

Parameter	Description	Value(s)
$\boldsymbol{a}$	Baseline per-capita host birth rate	0.11
$\boldsymbol{b}$	Host natural mortality rate	0.01
$\boldsymbol{c_H^1}$	Trade-off strength for hosts	0.05
$\boldsymbol{c_P^1}$	Trade-off strength for parasites	1
$\boldsymbol{c_H^2}$	Trade-off shape parameter for hosts	$\{-10,-3,3,10\}$
$\boldsymbol{c_P^2}$	Trade-off shape parameter for parasites	$\{-10,-3,3,10\}$
$\boldsymbol{d_{ij}}$	Number of effective resistance alleles	-
$\boldsymbol{G_{pr}}$	Metapopulation connectivity matrix	-
$\boldsymbol{G_{\Sigma p}}$	Number of connections for deme p	-
$\boldsymbol{L}$	Number of loci	4
$\boldsymbol{m_{ij}}$	Mutation matrix	-
$\boldsymbol{n_k}$	Number of types for species k	16
$\boldsymbol{q}$	Density-dependence on host births	0.001
$\boldsymbol{Q_{ij}}$	Susceptibility of host i to parasite j	-
$\boldsymbol{\alpha}$	Disease-associated mortality rate	0.05
$\boldsymbol{\beta}$	Transmission rate	$\{0.005, 0.01\}$
$\boldsymbol{\gamma}$	Recovery rate	0.05
$\boldsymbol{\mu_H}$	Host mutation rate	10^{-2}
$\boldsymbol{\mu_P}$	Parasite mutation rate	10^{-3}
$\boldsymbol{\rho}$	Dispersal rate per connection	5×10^{-5}
$\boldsymbol{\sigma}$	Reduction in susceptibility per effective resistance allele	0.2

Supplementary Table 4. Simulation analysis, showing the average difference (mean ± standard deviation over all simulations) between well and poorly connected populations. Positive values indicate higher average disease prevalence, resistance, or infectivity in the well-connected populations. Parameters as defined in Supplementary Table 3.

Factor	Parameter/type	Disease prevalence	Resistance	Infectivity
Network structure	Assortative	0.171±0.063	0.072±0.062	0.081±0.050
	Random	0.137±0.045	0.045±0.036	0.057±0.030
Transmissibility	$\beta = 0.005$	0.101±0.014	0.032±0.022	0.038±0.013
	$\beta = 0.01$	0.206±0.028	0.084±0.060	0.100±0.039
Host costs	$c_H^2 < 0$	0.155±0.049	0.094±0.051	0.073±0.043
	$c_H^2 > 0$	0.152±0.065	0.023±0.018	0.065±0.042
Parasite costs	$c_P^2 < 0$	0.155±0.058	0.058±0.053	0.050±0.024
	$c_P^2 > 0$	0.152±0.056	0.058±0.052	0.088±0.049
Overall	n/a	0.15±0.06	0.058±0.052	0.069±0.043

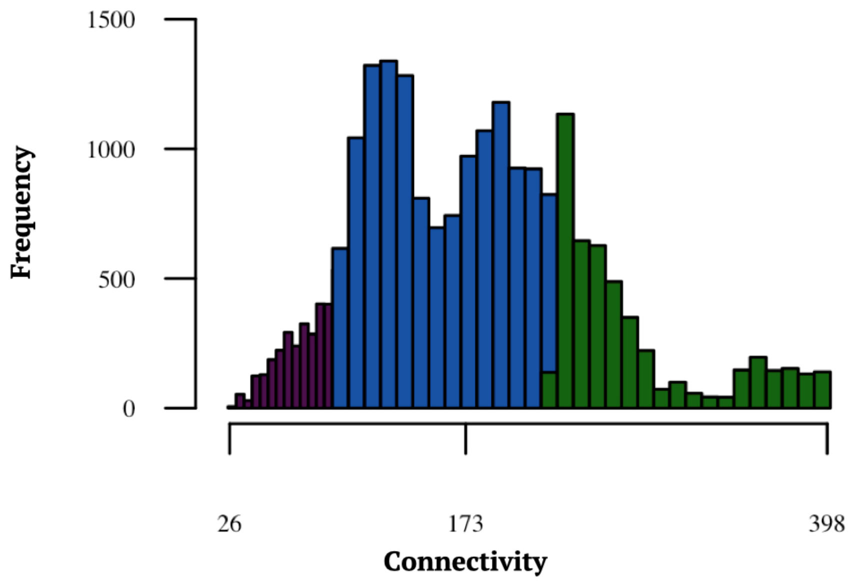

Supplementary Figure 1. The frequency of *P. lanceolata* populations in the three connectivity categories: low, intermediate and high. The categories were based on the 0.2 and 0.8 quantiles of the host connectivity (S^H) values showing the minimum, average and maximum values for S^H. Purple colour depict isolated populations, blue colour intermediate populations, and green colour well-connected populations. Source data are provided as a Source Data file.

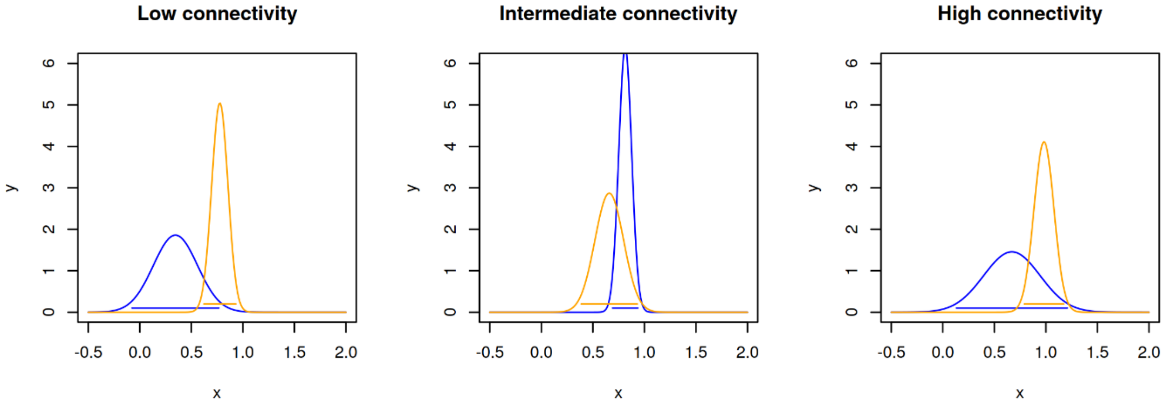

Supplementary Figure 2. The posterior distributions for the estimated effect of pathogen presence on the relative change in host population size for the three different connectivity. In each plot the effect posterior distribution for the effect of pathogen is shown in blue and its absence in yellow. Below the distributions the 95% credibility intervals are shown with the horizontal lines, while the modes of the distributions indicate the estimated mean effects. Source data are provided as a Source Data file.

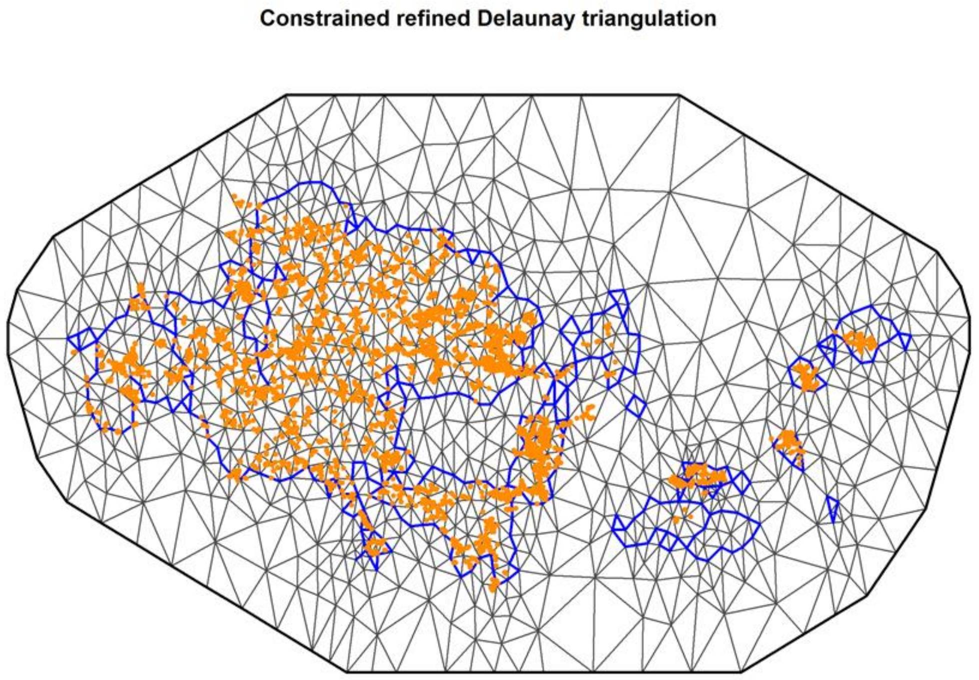

Supplementary Figure 3. The locations of host populations and the spatiotemporal mesh used for modelling the spatiotemporal random field. Orange color dots show the locations of *Plantago lanceolata* host populations in Åland islands, blue lines show the coastal line, and the dark grey mesh is used to fit the spatial model.

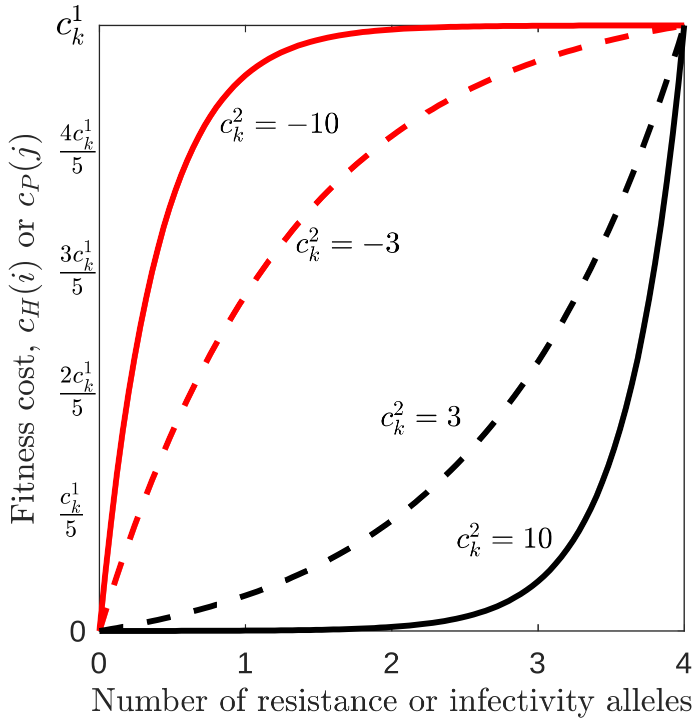

Supplementary Figure 4. Illustration of the fitness cost functions, $c_H(i)$ and $c_P(j)$, as described in the Materials and Methods. The maximum strength of the fitness cost, c_k^1 (for $k \in \{H, P\}$) corresponds to the maximum number of resistance or infectivity alleles (here, $L = 4$). The shape of the trade-off depends on the parameter c_k^2, with decelerating trade-offs occurring when $c_k^2 < 0$ (red) and accelerating trade-offs occurring when $c_k^2 > 0$ (black). The trade-offs accelerate/decelerate more strongly for larger values of $|c_k^2|$ (solid) compared to smaller values (dashed).
