# Supplementary material for: Spatially structured eco-evolutionary dynamics in a host-pathogen interaction render isolated populations vulnerable to disease
Source: Nat Commun. 2022 Oct 13;13:6018. doi: 10.1038/s41467-022-33665-3 (PMC9561709; doi:10.1038/s41467-022-33665-3)
Supplement: Supplementary file 3 — Reporting Summary [file 41467_2022_33665_MOESM3_ESM.pdf]

nature portfolio

Corresponding author(s): Anna-Liisa Laine

Last updated by author(s): May 9, 2022

Reporting Summary

Nature Portfolio wishes to improve the reproducibility of the work that we publish. This form provides structure for consistency and transparency in reporting. For further information on Nature Portfolio policies, see our Editorial Policies and the Editorial Policy Checklist.

Statistics

For all statistical analyses, confirm that the following items are present in the figure legend, table legend, main text, or Methods section.

n/a	Confirmed	
☐	☒	The exact sample size (*n*) for each experimental group/condition, given as a discrete number and unit of measurement
☒	☐	A statement on whether measurements were taken from distinct samples or whether the same sample was measured repeatedly
☐	☒	The statistical test(s) used AND whether they are one- or two-sided<br>*Only common tests should be described solely by name; describe more complex techniques in the Methods section.*
☐	☒	A description of all covariates tested
☐	☒	A description of any assumptions or corrections, such as tests of normality and adjustment for multiple comparisons
☐	☒	A full description of the statistical parameters including central tendency (e.g. means) or other basic estimates (e.g. regression coefficient) AND variation (e.g. standard deviation) or associated estimates of uncertainty (e.g. confidence intervals)
☐	☒	For null hypothesis testing, the test statistic (e.g. *F*, *t*, *r*) with confidence intervals, effect sizes, degrees of freedom and *P* value noted<br>*Give P values as exact values whenever suitable.*
☐	☒	For Bayesian analysis, information on the choice of priors and Markov chain Monte Carlo settings
☐	☒	For hierarchical and complex designs, identification of the appropriate level for tests and full reporting of outcomes
☒	☐	Estimates of effect sizes (e.g. Cohen's *d*, Pearson's *r*), indicating how they were calculated

Our web collection on statistics for biologists contains articles on many of the points above.

Software and code

Policy information about availability of computer code

Data collection	The data is available at https://github.com/ComputerBlue/SpatialEcoEvoDynamics
Data analysis	The analysis of host population growth rate was analysed using the INLA package (www.r-inla.org) in R version 4.0.1 and the code is available at https://github.com/ComputerBlue/SpatialEcoEvoDynamics. The custom code used for the simulation model is available at https://github.com/ecoevotheory/Hockerstedt_et_al_2022

For manuscripts utilizing custom algorithms or software that are central to the research but not yet described in published literature, software must be made available to editors and reviewers. We strongly encourage code deposition in a community repository (e.g. GitHub). See the Nature Portfolio guidelines for submitting code & software for further information.

Data

Policy information about availability of data

All manuscripts must include a data availability statement. This statement should provide the following information, where applicable:

- Accession codes, unique identifiers, or web links for publicly available datasets
- A description of any restrictions on data availability
- For clinical datasets or third party data, please ensure that the statement adheres to our policy

Data are available at https://github.com/ComputerBlue/SpatialEcoEvoDynamics

March 2021

Field-specific reporting

Please select the one below that is the best fit for your research. If you are not sure, read the appropriate sections before making your selection.

☐ Life sciences ☐ Behavioural & social sciences ☒ Ecological, evolutionary & environmental sciences

For a reference copy of the document with all sections, see nature.com/documents/nr-reporting-summary-flat.pdf

Life sciences study design

All studies must disclose on these points even when the disclosure is negative.

Sample size	*Describe how sample size was determined, detailing any statistical methods used to predetermine sample size OR if no sample-size calculation was performed, describe how sample sizes were chosen and provide a rationale for why these sample sizes are sufficient.*
Data exclusions	*Describe any data exclusions. If no data were excluded from the analyses, state so OR if data were excluded, describe the exclusions and the rationale behind them, indicating whether exclusion criteria were pre-established.*
Replication	*Describe the measures taken to verify the reproducibility of the experimental findings. If all attempts at replication were successful, confirm this OR if there are any findings that were not replicated or cannot be reproduced, note this and describe why.*
Randomization	*Describe how samples/organisms/participants were allocated into experimental groups. If allocation was not random, describe how covariates were controlled OR if this is not relevant to your study, explain why.*
Blinding	*Describe whether the investigators were blinded to group allocation during data collection and/or analysis. If blinding was not possible, describe why OR explain why blinding was not relevant to your study.*

Behavioural & social sciences study design

All studies must disclose on these points even when the disclosure is negative.

Study description	*Briefly describe the study type including whether data are quantitative, qualitative, or mixed-methods (e.g. qualitative cross-sectional, quantitative experimental, mixed-methods case study).*
Research sample	*State the research sample (e.g. Harvard university undergraduates, villagers in rural India) and provide relevant demographic information (e.g. age, sex) and indicate whether the sample is representative. Provide a rationale for the study sample chosen. For studies involving existing datasets, please describe the dataset and source.*
Sampling strategy	*Describe the sampling procedure (e.g. random, snowball, stratified, convenience). Describe the statistical methods that were used to predetermine sample size OR if no sample-size calculation was performed, describe how sample sizes were chosen and provide a rationale for why these sample sizes are sufficient. For qualitative data, please indicate whether data saturation was considered, and what criteria were used to decide that no further sampling was needed.*
Data collection	*Provide details about the data collection procedure, including the instruments or devices used to record the data (e.g. pen and paper, computer, eye tracker, video or audio equipment) whether anyone was present besides the participant(s) and the researcher, and whether the researcher was blind to experimental condition and/or the study hypothesis during data collection.*
Timing	*Indicate the start and stop dates of data collection. If there is a gap between collection periods, state the dates for each sample cohort.*
Data exclusions	*If no data were excluded from the analyses, state so OR if data were excluded, provide the exact number of exclusions and the rationale behind them, indicating whether exclusion criteria were pre-established.*
Non-participation	*State how many participants dropped out/declined participation and the reason(s) given OR provide response rate OR state that no participants dropped out/declined participation.*
Randomization	*If participants were not allocated into experimental groups, state so OR describe how participants were allocated to groups, and if allocation was not random, describe how covariates were controlled.*

Ecological, evolutionary & environmental sciences study design

All studies must disclose on these points even when the disclosure is negative.

Study description	We combine field surveys of infection and plant population size with a controlled laboratory inoculation study and a simulation model.
Research sample	The host population growth rate analysis is based on data obtained in the field on host population size (m2) and pathogen presence/

Research sample	absence (0/1). The number of populations in each category is provided in Table S2. The phenotyping assay used plants sampled from the natural populations as seeds and fungal spores collected from the field that were then purified and multiplied in the lab following a protocol described in detail in Laine, A.-L. (2004) Resistance variation within and among host populations in a plant-pathogen metapopulation – implications for regional pathogen dynamics. Journal of Ecology 92: 990-1000. doi: 10.1111/j.0022-0477.2004.00925.x
Sampling strategy	The field survey of infection is a long-term study where all known host population of P. lanceolata have been visited annually and surveyed for infection since year 2001. The search effort has been validated to be sufficient by carrying out control surveys of a subset of the same populations. The details of the metapopulation survey logistics are provided in Ojanen , S P , Nieminen , M , Meyke , E , Pöyry , J & Hanski , I 2013 , ' Long-term metapopulation study of the Glanville fritillary butterfly (Melitaea cinxia): survey methods, data management, and long-term population trends ' , Ecology and Evolution , vol. 3 , no. 11 , pp. 3713-3737 . https://doi.org/10.1002/ece3.733<br>The number of populations and plants within populations sampled for the inoculation study were determined based on prior inoculation studies of this system that have shown this sampling effort to be able to detect within vs. among population differences in resistance<br>Laine, A.-L. (2004) Resistance variation within and among host populations in a plant-pathogen metapopulation – implications for regional pathogen dynamics. Journal of Ecology 92: 990-1000. doi: 10.1111/j.0022-0477.2004.00925.x<br>Laine, A.-L. (2005) Spatial scale of local adaptation in a plant-pathogen metapopulation. Journal of Evolutionary Biology 18:930-938. doi: 10.1111/j.1420-9101.2005.00933.x<br>Laine, A.-L. (2008) Temperature-mediated patterns of local adaptation in a natural plant-pathogen metapopulation. Ecology Letters 11:327-337. doi: 10.1111/j.1461-0248.2007.01146.x
Data collection	Each visited host population was visually surveyed for signs of infection during the study period. Each year 40 undergraduate biology students survey the Åland metapopulation system estimating P. lanceolata population size as m2 and presence absence of the fungal pathogen P. plantaginis. A leaf sample of infection was always collected for microscopy to confirm the pathogen species identification. In the field data is collected using the Earthcape software (https://earthcape.com) using portable PCs and tables. The survey is described in detail in Ojanen , S P , Nieminen , M , Meyke , E , Pöyry , J & Hanski , I 2013 , ' Long-term metapopulation study of the Glanville fritillary butterfly (Melitaea cinxia): survey methods, data management, and long-term population trends ' , Ecology and Evolution , vol. 3 , no. 11 , pp. 3713-3737 . https://doi.org/10.1002/ece3.733.<br>The seeds and fungal samples for the phenotyping assay were collected by L. Höckerstedt. Seed were collected and stored in paper envelopes until they were sown in the greenhouse on Viikki campus, University of Helsinki. The pathogen was sampled as infected leaves onto patri dishes with a moist filter paper, and stored at + 5 C until taken to the lab were fungal spored were inoculated onto fresh P. lanceolata leaves.
Timing and spatial scale	The field survey data was collected between 2001-2008, the study area is appr. 50 x 80 km. Samples for the phenotyping assay were collected in August 2014.
Data exclusions	No data were excluded
Reproducibility	Each inoculation was replicated twice. If there was discrepancy between the replicates, interaction was always as 'infection' if infection established in either trial. This is described in the manuscript.
Randomization	Leaves were randomized among the petri dishes in the inouclation experiment.
Blinding	Scoring of infection was done without knowledge of plant or pathogen source

Did the study involve field work? ☒ Yes ☐ No

Field work, collection and transport

Field conditions	The field work was carried out across some thousands of local populations of Plantago lanceolata in September of each year between 2001-2008.´
Location	Åland archipelago, Finland
Access & import/export	Each local population is briefly visited. Finnish legislation allows visiting also private land and collecting samples without any permits. However, if a local population was within a visible distance from housing, a permission to survey the site was always requested. The survey has been carried out in this area for 30 years, locals are very familiar with the work and are positive about it.
Disturbance	The study populations are located typically in immediate proximity of housing and agricultural practices, and hence they are subjected to regular human impact. Our annual field survey of infection that involves two field assistants walking through each habitat patch and sampling one P. lanceolata leaf is expected to have negligible impact on this ecosystem. Likewise, sampling of seed and some infected leaves for the phenotyping assay is not expected have any impact on the system.

Reporting for specific materials, systems and methods

We require information from authors about some types of materials, experimental systems and methods used in many studies. Here, indicate whether each material, system or method listed is relevant to your study. If you are not sure if a list item applies to your research, read the appropriate section before selecting a response.

Materials & experimental systems

n/a	Involved in the study	
☒	☐	Antibodies
☒	☐	Eukaryotic cell lines
☒	☐	Palaeontology and archaeology
☐	☒	Animals and other organisms
☒	☐	Human research participants
☒	☐	Clinical data
☒	☐	Dual use research of concern

Methods

n/a	Involved in the study	
☒	☐	ChIP-seq
☒	☐	Flow cytometry
☒	☐	MRI-based neuroimaging

Animals and other organisms

Policy information about studies involving animals; ARRIVE guidelines recommended for reporting animal research

Laboratory animals	*For laboratory animals, report species, strain, sex and age OR state that the study did not involve laboratory animals.*
Wild animals	*Provide details on animals observed in or captured in the field; report species, sex and age where possible. Describe how animals were caught and transported and what happened to captive animals after the study (if killed, explain why and describe method; if released, say where and when) OR state that the study did not involve wild animals.*
Field-collected samples	Field collected leaves and seed of Plantago lanceolata, some infected with powdery mildew fungus. No permits are required to do this.
Ethics oversight	There are no ethical concerns related to this.

Note that full information on the approval of the study protocol must also be provided in the manuscript.
